# Importance of History in the Management of New-Onset Hyperkalemia

**DOI:** 10.7759/cureus.106682

**Published:** 2026-04-08

**Authors:** Igor Kagan, Kristine Sarmosyan, Sean Lei, Michelle Hwang

**Affiliations:** 1 Division of Nephrology, University of California Los Angeles David Geffen School of Medicine, Los Angeles, USA

**Keywords:** drug-induced hyperkalemia, electrolyte abnormalities, general nephrology, hyperkalemia management, timolol eye drops

## Abstract

Hyperkalemia is a life-threatening disorder with a multitude of causes. We present a case of a 64-year-old woman with new-onset hyperkalemia. A patient with a documented history of normokalemia developed consistent hyperkalemia on repeat blood testing. The patient was initially managed by her primary care physician, then referred to nephrology for persistent hyperkalemia, at which point sodium polystyrene sulfonate was initiated. The patient presented for a second opinion on the hyperkalemia, and after a thorough history and chart review, it was determined that the patient’s dorzolamide-timolol eye drops were the cause of the hyperkalemia. Discontinuation of the eye drops resolved the hyperkalemia.

## Introduction

Hyperkalemia is an electrolyte disorder where the measured serum potassium is typically over 5.3 mmol/L. Severe hyperkalemia can be lethal, and thus, it is important to quickly diagnose and treat the electrolyte disorder [1]. The etiology of hyperkalemia is extensive, including both genetic and acquired causes [2]. Genetic causes are typically noted early in life, while acquired causes can arise from a myriad of reasons [3]. The differential diagnosis for hyperkalemia includes kidney failure, diabetes mellitus, adrenal disease, and medications such as angiotensin-converting enzyme (ACE) inhibitors, angiotensin receptor blockers, potassium-sparing diuretics, and beta-blockers [3]. It is estimated that medication-induced hyperkalemia contributes to over 60% of hyperkalemia cases [4]. Medication-induced hyperkalemia can be due to changes to transmembrane potassium movement [5]. For new-onset hyperkalemia in an adult, it is important to conduct a full evaluation to uncover the etiology of the hyperkalemia. As this case demonstrates, a detailed history is important in helping diagnose the cause of the hyperkalemia.

## Case presentation

**Figure 1:**
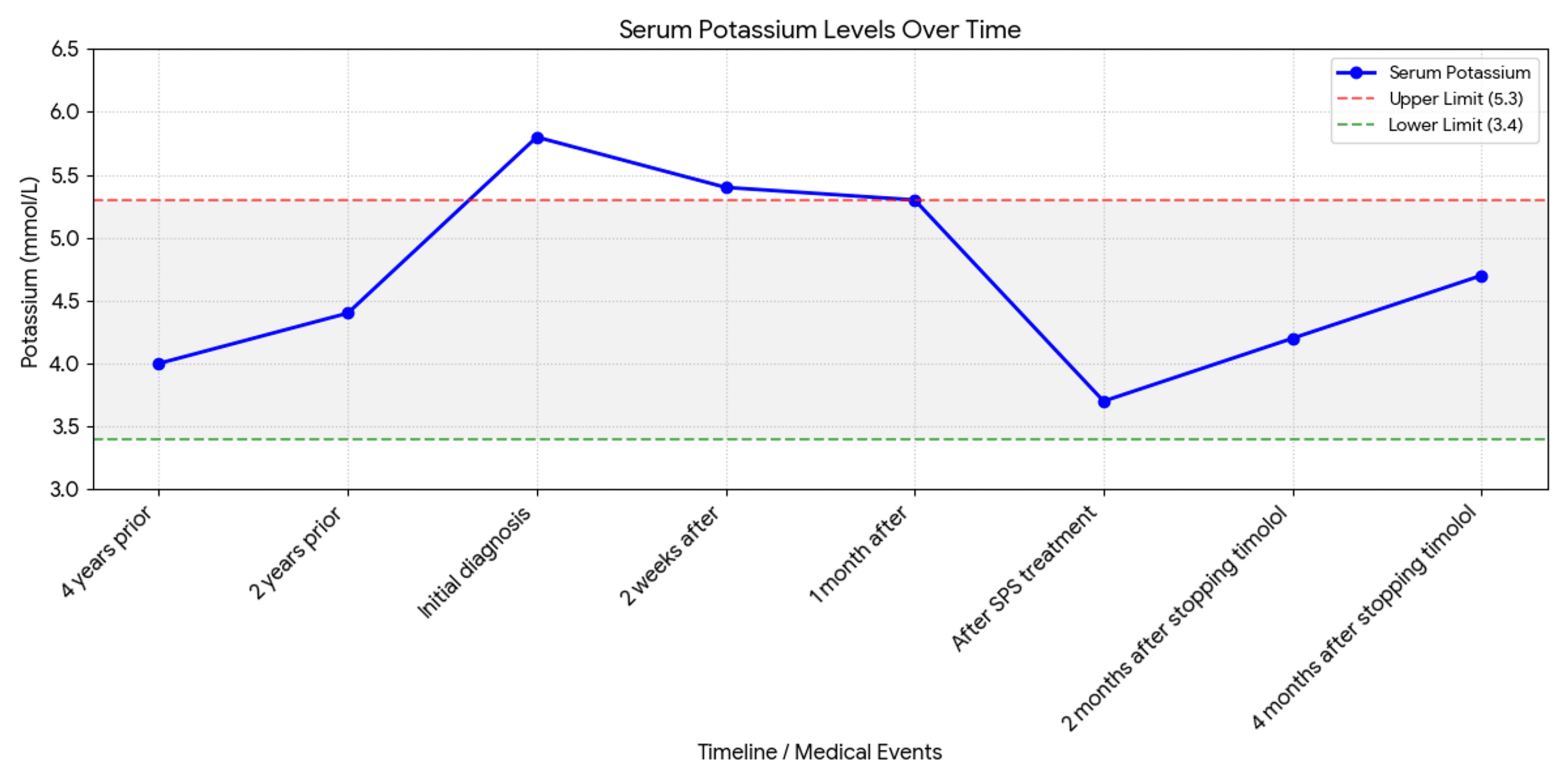
Trendline for Potassium SPS: sodium polystyrene sulfonate

A 64-year-old female patient with a past medical history of glaucoma was found to have hyperkalemia of 5.8 mmol/L during her primary care visit (Figure 1). Potassium remained elevated at 5.4 mmol/L upon repeat testing. The patient was counseled to discontinue alkaline water consumption that was supplemented with potassium. The follow-up potassium level a few weeks later came back 5.3 mmol/L. Given the persistent hyperkalemia, the patient was referred to nephrology for further management, where she was started on sodium polystyrene sulfonate for treatment of hyperkalemia. Her serum potassium improved to 3.7 mmol/L. However, the patient was hesitant to continue long-term therapy with sodium polystyrene sulfonate and presented to the University of California Los Angeles (UCLA) nephrology for a second opinion. A detailed review of the patient’s history confirmed that hyperkalemia was a new issue. Her prior potassium was 4.0 mmol/L four years prior and 4.4 mmol/L two years prior to presentation. At that time, she had normal kidney function with a serum creatinine of 0.8 mg/dL. She denied any changes in diet or bowel habits. Notably, she reported starting a new ophthalmic medication, dorzolamide-timolol, for her glaucoma treatment. Beta-blockers can result in hyperkalemia, and prior case reports confirm that the use of timolol eye drops can result in hyperkalemia [6]. In coordination with ophthalmology, the patient was transitioned from dorzolamide-timolol to latanoprost. Following this change, sodium polystyrene sulfonate was discontinued, and repeat laboratory testing confirmed sustained resolution of hyperkalemia on two separate measurements.

## Discussion

The differential diagnosis for hyperkalemia is extensive and can be broken down into increased endogenous versus exogenous potassium and also causes of dysregulation of aldosterone and renin [7]. Addison's disease (primary adrenal insufficiency), which can result from genetic causes or develop later in life from autoimmune disease, was unlikely in this patient. The patient had no prior history of hyperkalemia until now in her 60s, thus effectively ruling out genetic causes. She also did not report any new symptoms to signify an underlying autoimmune disorder. Other causes of endogenous hyperkalemia, such as tissue necrosis, tumor lysis, or hemolysis, were ruled out, given the patient’s chronic benign presentation. The patient denied any diet changes or utilization of salt substitutes that can be potassium-rich, thus making a new exogenous process unlikely. No bowel changes were repeated [8].

Pseudohyperkalemia due to hemolysis and excessive leakage of potassium from cells [9] was unlikely, as the patient had several confirmed measurements of hyperkalemia before she was started on treatment. Additionally, her complete blood count was normal, ruling out pseudohyperkalemia from leukocytosis and thrombocytosis [9]. There was no history of diabetes, and serum glucose levels were normal, making insulin deficiency unlikely. Serum bicarbonate was also normal, making metabolic acidosis another unlikely cause.

After a detailed evaluation of the laboratory testing, ruling out the possible causes listed above, the evaluation involved an extensive patient history to identify any new triggers. Medications are a common cause of hyperkalemia [10]. Common classes of medication that result in hyperkalemia include potassium-sparing diuretics, ACE inhibitors, angiotensin-II blockers, immunosuppressive medications such as cyclosporine and tacrolimus, anticoagulation medication heparin, nonsteroidal anti-inflammatory drugs (NSAIDs), and beta-blockers. Potassium-sparing diuretic spironolactone results in hyperkalemia due to being an aldosterone antagonist. Triamterene, pentamidine, and amiloride block sodium channels in principal cells. NSAIDs, ACE inhibitors, and angiotensin-II receptor blockers result in decreased aldosterone synthesis along with decreased renal blood flow and glomerular filtration rate. Beta-blockers decrease beta-2-driven potassium uptake, leading to hyperkalemia. The medication review for our patient was straightforward, as she was only on one medication. The patient started dorzolamide-timolol for glaucoma shortly before the onset of hyperkalemia. A case report from 1986 [6] described a 72-year-old man with no prior history of hyperkalemia who developed hyperkalemia after starting timolol, which resolved after discontinuation and recurred upon rechallenge. Another case report described a four-month-old infant receiving timolol maleate 0.5% ophthalmic solution [11]. The infant developed hyperkalemia, which resolved after stopping the ophthalmic solution. Similar to our case, the patient’s hyperkalemia resolved once timolol was discontinued. Her serum potassium remained 4.2 and 4.7 mmol/L two and four months after timolol discontinuation, respectively.

## Conclusions

This case demonstrates the importance of critical thinking and careful review of the patient’s history in arriving at the appropriate diagnosis. Our patient had no history of hyperkalemia, then suddenly presented to her primary doctor with confirmed hyperkalemia on numerous blood work results. The patient was started on treatment with sodium polystyrene sulfonate for the hyperkalemia. When the patient presented for a second opinion and a full history was obtained, it became evident that a medication was likely the cause of the hyperkalemia. This case demonstrates the importance of uncovering the cause of new-onset hyperkalemia. Establishing the correct diagnosis allowed the patient to discontinue sodium polystyrene sulfonate and eliminated the ongoing concern for hyperkalemia and frequent laboratory testing.
